# Effects and associated transcriptomic landscape changes of methamphetamine on immune cells

**DOI:** 10.1186/s12920-022-01295-9

**Published:** 2022-06-28

**Authors:** Deshenyue Kong, Jun-Hong Mao, Hong Li, Jian-Yu Wang, Yu-Yang Li, Xiao-Cong Wu, Guo-Fen Re, Hua-You Luo, Yi-Qun Kuang, Kun-Hua Wang

**Affiliations:** 1 NHC Key Laboratory of Drug Addiction Medicine, Kunming Medical University, Kunming, 650032 China; 2 Yunnan University, Kunming, 650032 China; 3 Narcotics Control Bureau of the Ministry of Public Security of Yunnan Province, Kunming, 650032 China; 4 Scientific Research Laboratory Center, First Affiliated Hospital of Kunming Medical University, Kunming, 650032 China; 5 Department of Gastrointestinal and Hernia Surgery, First Affiliated Hospital of Kunming Medical University, Kunming, 650032 China

**Keywords:** Methamphetamine, Immune cell, Transcriptome, Apoptosis, Cholesterol metabolic, Phospholipid metabolic

## Abstract

**Background:**

Methamphetamine (METH) abuse causes serious health problems, including injury to the immune system, leading to increased incidence of infections and even making withdrawal more difficult. Of course, immune cells, an important part of the immune system, are also injured in methamphetamine abuse. However, due to different research models and the lack of bioinformatics, the mechanism of METH injury to immune cells has not been clarified.

**Methods:**

We examined the response of three common immune cell lines, namely Jurkat, NK-92 and THP-1 cell lines, to methamphetamine by cell viability and apoptosis assay in vitro, and examined their response patterns at the mRNA level by RNA-sequencing. Differential expression analysis of two conditions (control and METH treatment) in three types of immune cells was performed using the DESeq2 R package (1.20.0). And some of the differentially expressed genes were verified by qPCR. We performed Gene Ontology and Kyoto Encyclopedia of Genes and Genomes analysis of differentially expressed genes by the clusterProfiler R package (3.14.3). And gene enrichment analysis was also performed using MetaScape (www.metascape.org).

**Results:**

The viability of the three immune cells was differentially affected by methamphetamine, and the rate of NK-cell apoptosis was significantly increased. At the mRNA level, we found disorders of cholesterol metabolism in Jurkat cells, activation of ERK1 and ERK2 cascade in NK-92 cells, and disruption of calcium transport channels in THP-1 cells. In addition, all three cells showed changes in the phospholipid metabolic process.

**Conclusions:**

The results suggest that both innate and adaptive immune cells are affected by METH abuse, and there may be commonalities between different immune cells at the transcriptome level. These results provide new insights into the potential effects by which METH injures the immune cells.

**Supplementary Information:**

The online version contains supplementary material available at 10.1186/s12920-022-01295-9.

## Introduction

Drug use is one of the contributors to the global burden of disease [1]. According to the World Drug Report 2021, approximately 275 million people worldwide used drugs in 2020 [2]. The healthcare system faces an increasingly difficult task. Deaths related to drug use disorders have almost doubled in the last decade. Methamphetamine (METH) is a highly addictive psychostimulant that is gaining popularity among drug users worldwide. In 2019, there were an estimated 27 million amphetamine users, equivalent to 0.5% of the global population aged 15–64 years, already causing a major global public health problem [2].

It has been shown that methamphetamine causes innate and adaptive immune impairment and increases the incidence of infectious diseases [3]. METH use may be associated with human immunodeficiency viruses (HIV) and hepatitis C virus (HCV) infection [4–6], mainly caused by sharing syringe needles [1]. However, increased susceptibility due to METH-induced immune depression still cannot be excluded. In addition, studies showed that METH use was also associated with methicillin-resistant staphylococcus aureus skin infections [7], tuberculosis transmission [8], cryptococcus neoformans pulmonary infection and dissemination to the brain. Immune cells, the most important component of the immune system, may be impaired during METH use. However, the mechanism of immune cell impairment has not been fully described due to differences in research models and methods. In a study of the METH dose-escalation mouse model, the number and frequency of CD4^+^ and CD8^+^T-cell subsets, nature killing (NK) cells, dendritic cells (DCs) and monocytes/macrophages were reduced [9]. However, another study showed that the frequency of METH administration and the sex difference all contributed to the different immune responses. Single and repeated METH injections reduced NK-cell activity especially in female mice with 5 injections, but 10 injections recovered them to the level of controls [10].

Different METH treatments may lead to different responses. A low concentration of METH can already cause T-cell dysfunction. For example, 25 μM METH treatment decreased the expression of T-cell surface molecules CD3 and CD28, as well as the level of IL-2 production [11]. Most studies have used METH concentrations of 100 μM and the results have mostly shown T-cell injury, such as oxidative stress and mitochondrial injury [3], cell cycle altered, and impaired proliferation [12]. In addition, METH was found to enhance HIV replication in T cells [13–15]. Interestingly, the opposite effect was observed for METH concentrations above 100 μM, which inhibited HIV-1 replication [16]. Moreover, treatment with a high concentration of METH at 2 mM resulted in significant T-cell apoptosis [17]. In studies on NK cells, the results showed that they were significantly activated by METH, accompanied by a decrease in cell numbers and dysfunction [9, 18]. A majority of studies on METH-induced immune responses in monocytes/macrophages focused on the process of HIV infection. Results suggested that METH promoted HIV replication in monocytes/macrophages [19, 20]. Furthermore, as a weak base, METH led to acidic organelle injury, and disrupted the pH gradient, leading to immune cell dysfunction [21, 22].

Although many studies on METH-mediated immune cell responses have been reported [12, 23, 24], most of them were discussed in the context of co-morbidities with other diseases, such as HIV infection. In addition, the lack of bioinformatic analysis has led to the limitations of these studies [24]. To better understand METH-mediated innate and adaptive immune cell responses, we evaluated the effects of METH on resting-state immune cell responses and transcriptome responses through three METH abuse cell models in vitro. The results provide a better understanding of the molecular targets by which human immune cells respond to METH abuse.

## Methods

### Drugs

METH was provided by Yunnan Public Security Bureau, and its purity was tested in Yuxi Police Judicial Expertise Center. It was dissolved in phosphate-buffered saline (PBS; Biological Industries) to a concentration of 10 mM and stored away from light at − 20 °C.

### Cells culture

We conducted in vitro studies on cell lines by using three common human immune cell models, namely Jurkat, NK-92, and THP-1. The Jurkat cell line is derived from human T-cell leukemia and is always used as a model of T cell signaling [25]. The NK‐92 cell line is derived from a non‐Hodgkin's lymphoma and is commonly used for functional studies of natural killer cells [26]. And THP-1 cells are a human leukemic monocyte line commonly used to study monocyte/macrophage-related functions and mechanisms [27]. Jurkat and THP-1 (Kunming Cell Bank of Chinese Academy of Sciences) cells were cultured in RPMI Medium 1640 (Invitrogen) supplemented with 10% fetal bovine serum (Biological Industries). NK-92 (Procell, Wuhan, China) cells were cultured in MEMα (PM150422) with 0.2 mM Inositol, 0.1 mM β-mercaptoethanol (PB180633), 0.02 mM Folic Acid, 200U/mL recombinant IL-2, 12.5% HS (164215-100), 12.5% FBS (164210-500), and 1% P/S (PB180120) (Procell, Wuhan, China). All cell lines were used at less than passage 10. Exponentially growing cells, at approximately 60–75% confluence, were counted, and seeded into T25 culture flasks (5.0 × 10^5^ cells/flask, 25 cm^2^ growth area) (Corning). The cells were incubated at 37 °C in a 5% CO_2_ humidified incubator.

### Cell viability assay

Cell viability assay was performed by Cell Counting Kit 8 (CCK-8) (Dojindo). 100 μl of a culture medium containing 5 × 10^4^ Jurkat or THP-1 cells were added to each well of a 96-well plate, and 3 × 10^4^ NK-92 cells were added to each well of a 96-well plate. Cells were then incubated at 37 °C in a 5% CO_2_ humidified incubator with METH in a concentration gradient of 0 μM, 100 μM, 250 μM, 500 μM, 1000 μM, and 2000 μM for 12 h, 24 h, 48 h, and 72 h, respectively. Then CCK-8 solution (10 μl per 100 μl of the medium in each well) was added to each well and incubated for an additional 2 h. And then the absorbance at 450 nm was measured. Each time point and METH concentration was repeated in four wells and the experiment was independently performed for three times at least.

### Apoptosis studies

Apoptosis detection was performed with the Annexin V-FITC Apoptosis Detection Kit (Beyotime, Shanghai, China). In brief, 2 ml of a culture medium containing 5 × 10^5^/ml Jurkat or THP-1 cells were added to each well of a 6-well plate, and 3 × 10^4^/ml NK-92 cells were added to each well of a 6-well plate. Cells were then incubated at 37 °C in 5% CO_2_ in a humidified environment with METH in a concentration gradient of 0 μM, 250 μM, 500 μM, and 1000 μM for 12 h, 24 h, and 48 h, respectively. Then we collected cells and washed them with PBS twice and then resuspended them in 195 μL binding buffer. 5 μL Annexin V-FITC solution was added to the cells and incubated for 10 min at room temperature in the dark. Then the cells were resuspended in 190 μL binding buffer after centrifugation. Next 10 μL propidium iodide (PI) was added to the cells and then analyzed by flow cytometry using NovoCyte™ flow cytometer (NovoCyte 2060R; ACEA Bioscience, Inc.). We analyzed the data by FlowJo software (Tree Star, Ashland, OR, USA). Independently, the experiment was performed for three times at least.

### RNA sequencing

#### Sample collection and preparation

##### RNA quantification and qualification

Total RNA was isolated from Jurkat cells, THP-1 cells and NK-92 cells and their METH-treated derivatives with 250 μM for 24 h respectively using a Trizol RNA isolation kit (Qiagen). Total amounts and integrity of RNA were assessed using the RNA Nano 6000 Assay Kit of the Bioanalyzer 2100 system (Agilent Technologies, CA, USA).

##### Library preparation for Transcriptome sequencing

Total RNA was used for the RNA sample preparations. Briefly, we used poly-T oligo-attached magnetic beads to purify the mRNA. Fragmentation was performed at elevated temperature using divalent cations in First Strand Synthesis Reaction Buffer (5 ×). First-strand cDNA was synthesized using random hexamer primer and M-MuLV Reverse Transcriptase, then the RNA was degraded by RNaseH. Second strand cDNA was synthesized using DNA Polymerase I and dNTP. By exonuclease/polymerase activities, the remaining overhangs were converted into blunt ends. After 3’ ends of DNA fragments adenylation, Adaptor with hairpin loop structure was ligated and ready for hybridization. To preferentially select cDNA fragments with lengths of 370 ~ 420 bp, the library fragments were purified with AMPure XP system (Beckman Coulter, Beverly, USA). PCR amplification was then performed and the PCR products were purified using AMPure XP beads to obtain the library.

After the library construction, the library was initially quantified using a Qubit2.0 Fluorometer, then diluted to 1.5 ng/μl, and the library insert size was detected using an Agilent 2100 bioanalyzer. After the insert size was as expectation, qRT-PCR was used to accurately quantify the library effective concentration (higher than 2 nM) to ensure the library quality.

##### Clustering and sequencing

After the library is qualified, the different libraries are pooled according to the effective concentration and the target amount of data off the machine, then sequenced by the Illumina NovaSeq 6000.

#### Data analysis

##### Quality control

Clean data were obtained by removing reads with adapter, N base and low-quality from the raw data. And Q20, Q30, and GC content of the clean data were also calculated. All subsequent analyses were based on high quality clean data.

##### Reads mapping to the reference genome

Reference genome and gene model annotation files were downloaded from the genome website directly. Index of the reference genome was built using Hisat2 (v2.0.5) and paired-end clean reads were aligned to the reference genome using Hisat2 (v2.0.5).

##### Differential expression analysis

Differential expression analysis of two conditions (control and METH treatment) in three types of immune cells (three biological replicates per condition) was performed using the DESeq2 R package (1.20.0). DESeq2 provides statistical routines for determining differential expression in digital gene expression data using a model based on the negative binomial distribution. *p* < 0.05 and |log2(foldchange)|> 0.5 were set as the threshold for significantly differential expression.

##### Enrichment analysis of differentially expressed genes

Gene Ontology (GO) enrichment analysis of differentially expressed genes was implemented by the clusterProfiler R package (3.14.3), in which gene length bias was corrected. GO terms with corrected *p* value less than 0.05 were considered significantly enriched by differential expressed genes. Kyoto Encyclopedia of Genes and Genomes (KEGG) is a database resource for understanding high-level functions and utilities of the biological system (http://www.genome.jp/kegg/) [28]. We used the clusterProfiler R package (3.14.3) to test the statistical enrichment of differential expression genes in KEGG pathways. In addition, we also used Metascape (http://metascape.org) for gene enrichment analysis, which includes GO biological processes, KEGG signaling pathways, and Reactome pathways. Data visualization was performed using the ggplot2 package (3.3.3) [29].

##### Protein–protein interaction analysis of differentially expressed genes

Protein–protein interaction (PPI) analysis was based on the STRING database (http://string-db.org/). PPI network was obtained by Cytoscape (version 3.2.0; Cytoscape Consortium). According to node connectivity, genes were further identified as hub genes with the MCC algorithm, using cytoHubba package/Cytoscape software [30, 31].

Gene expression data is available under GEO (Gene Expression Omnibus) accession number GSE201007.

### Validation of the RNA sequencing profiles by quantitative real-time PCR

To validate the differentially expressed genes identified through RNA sequencing (RNA-seq), 10 genes (*MVD*, *HMGCS1*, *FADS2*, *LSS*, *DHCR24*, *S1PR2*, *CCL1*, *FCER1G*, *PLCB4*, *PLD1*) were selected for quantitative real-time PCR (qPCR). To avoid the batch effect and strengthen the validity, we used the same RNA samples prepared for RNA sequencing. Gene-specific qPCR primers were designed using Primer Premier 6.0 software (Premier Biosoft, Palo Alto, CA, United States) (Table 1), and the efficiency was evaluated with an amplification plot and a melting curve. And *GAPDH* gene was used as a housekeeping gene. Reverse transcription of total RNA into cDNA was performed using the Goldenstar RT6 cDNA Synthesis Mix (Tsingke Biotechnology, Beijing, China), following the manufacturer’s protocol. Finally, qPCR was conducted using 2 × T5 Fast qPCR Mix (SYBR Green I) (Tsingke Biotechnology, Beijing, China) and FQD-96A system (Bioer, Hangzhou, China). For each sample, qPCR reactions were performed in triplicate, and the average CT was calculated. The CT values of the different samples were compared using the 2^−ΔΔCT^ method. Experiments were performed using three distinct biological replicates per group.

**Table 1:** Primers used in reverse transcription-quantitative real-time PCR

Gene	Primer sequence	
	5′–3′ Forward	5′–3′ Reverse
*MVD*	ACCTTCCCGCCCATCTCTTA	AACTCAGCCACAGTGTCGTC
*HMGCS1*	GGCACCAGATGTCTTCGCT	CTTTCTTGGCAGGGCTTGGA
*FADS2*	CTGCTGATTGGTGAACTGGC	ACAGGTTCATGTCCTCAGCC
*LSS*	ACTGGACGGGTGATTATGGTG	TGCACTGACCGCAGGTA
*DHCR24*	TCTTCCGCTACCTCTTTGGC	ATGAACGGACACAGCCAGAT
*S1PR2*	CTCTCGGCCTCTGTCTTCAG	TCACCACGCACAGCACATAA
*CCL1*	CTCCATCTGCTCCAATGAGGG	GGAATGGTGTAGGGCTGGTA
*FCER1G*	CTCCAGCCCAAGATGATTCCA	TCGACAGTAGAGGAGGGTGAG
*PLCB4*	GAAAACGCCCCAGTCTTCCT	AACACATCTGCAACCAGCCA
*PLD1*	CCTCCAATACCGGGTCCATC	GGCGTGGAGTACCTGTCAAT
*GAPDH*	AGGTCGGTGTGAACGGATTTG	GGGGTCGTTGATGGCAACA

### Statistical analyses

All analyses were performed using GraphPad Prism 9.0 (GraphPad Prism Software, Inc.). Results were presented as mean ± SEM. An unpaired, two-tailed Student’s test was used for comparisons between two groups. Data of multiple groups were compared using one-way ANOVA, followed by Tukey’s multiple comparisons test. *p* < 0.05 was indicated statistical significance.

## Results

### METH inhibited immune cell viability

**Fig. 1:**
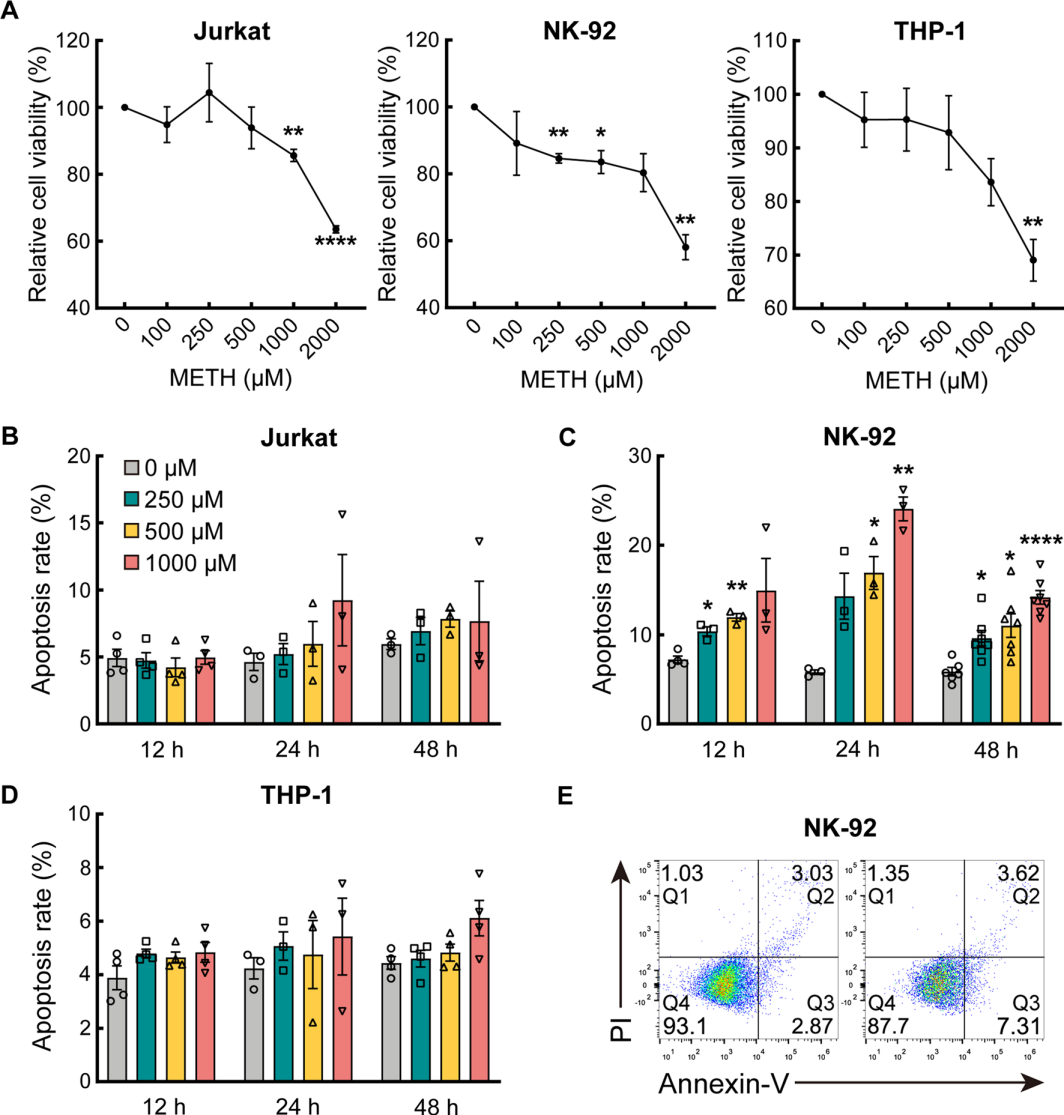
Methamphetamine inhibited immune cell viability and promoted apoptosis. **a** CCK-8 assay was used to assess Jurkat cell, NK-92 cell and THP-1 cell viability after treatment with different METH concentrations at 24 h. Data represent mean ± SEM from three to seven independent experiments. **b**–**d** Quantitative results of the apoptosis cell percentages. Annexin V-FITC/PI analysis was used to assess Jurkat cell (**b**), NK-92 cell (**c**) and THP-1 cell (**d**) apoptosis after treatment with different METH concentrations and different treatment times by flow cytometry. **e** Flow cytometry analysis unveiled the apoptosis rates of the NK-92 cells treated with 250 μM METH at 24 h. Cell apoptosis includes early (Q3) and late (Q2) apoptosis. Percentage of cell apoptosis = Q2 + Q3. Apoptosis rates are displayed as histograms. Each bar represents the mean ± SEM (*p < 0.05; **p < 0.01; ***p < 0.001; ****p < 0.0001, compared with 0 μM)

Within the general trend, CCK-8 revealed that METH inhibited the cell viability of all three immune cells at higher concentrations. 0–250 μM METH showed mild viability inhibitory activity. In Jurkat and NK-92, 1000 μM METH treatment exhibited toxicity, however, 2000 μM METH treatment showed toxicity to THP-1 (cell viability < 80%) (Additional file 1: Figure S1). Treatment with 250 μM METH for 24 h did not reveal significant inhibitory effects in all three cells (cell viability > 80%) (Fig. 1a). Accordingly, the optimal METH concentration for subsequent experiments was chosen as 250 μM, and the treatment time was 24 h.

### METH led to apoptosis in NK-92 cells

Apoptosis of immune cells is important in the development of immune function impairment. We found apoptotic cells shrink, reduced cell clump formation, and membrane blebbing (results not shown). Dot plots of flow cytometry showed the frequency of apoptotic cells. The Q1 quadrant represents the dead cells; the Q2 quadrant represents the late apoptotic cells; the Q3 quadrant represents the early apoptotic cells; and the Q4 quadrant represents the living cells. We found that METH led to a significantly higher rate of apoptosis in NK cells compared to the control group (Fig. 1c, e). There was a trend of increased apoptosis in the other two cell types (Fig. 1b, d). The complete results were shown in Additional file 2: Figure S2.

### Transcriptomic response of immune cells treated with METH

**Fig. 2:**
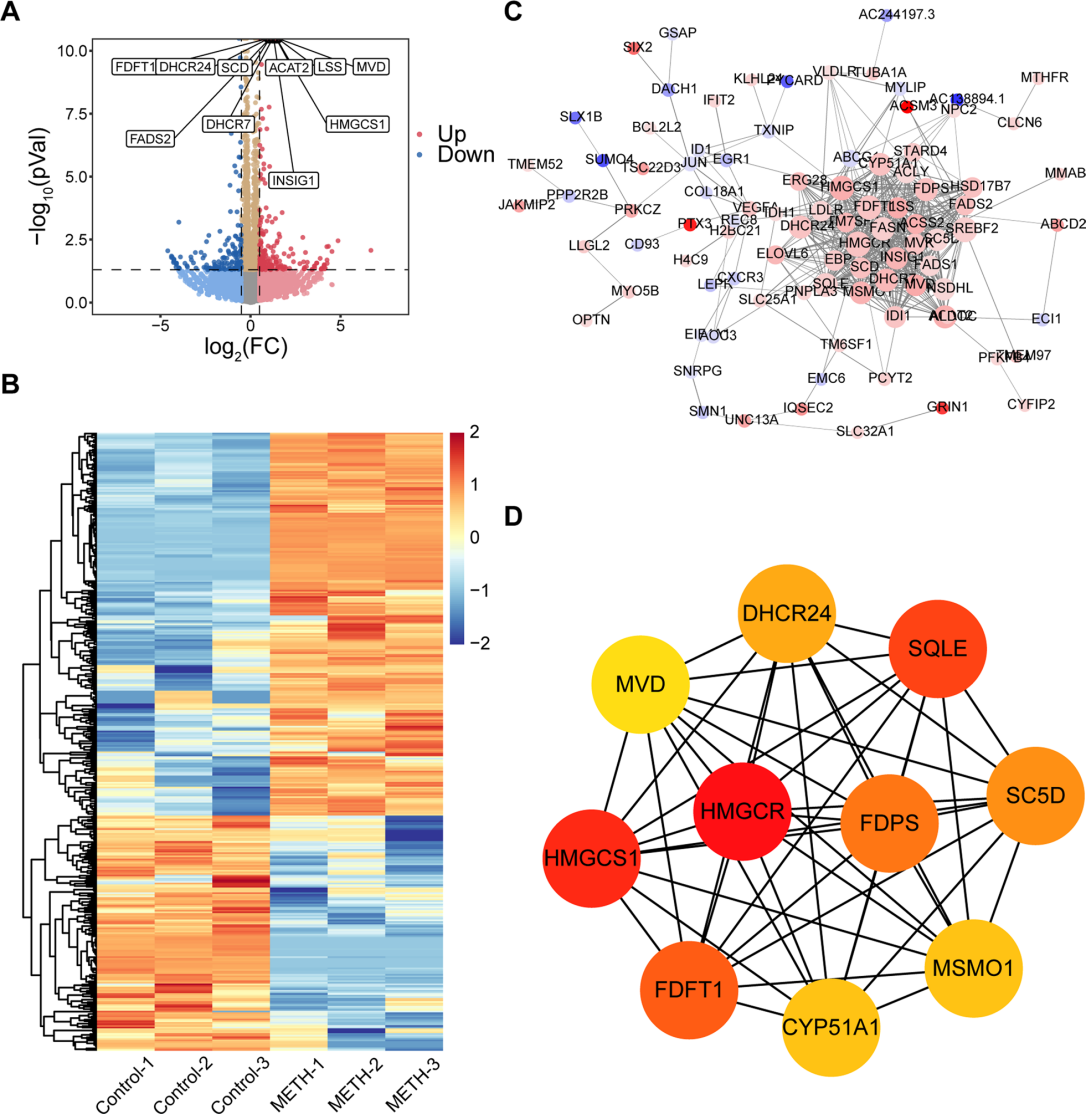
Identification of differentially expressed genes (DEGs) in Jurkat cells with methamphetamine treatment. **a** Volcano plot showing DEGs (|Log_2_(foldchange)|> 0.5 and *p* < 0.05) between control (n = 3) and METH-treated cells (n = 3). Top 10 DEGs sorted by *p* value and genes of interest were shown in the plot. **b** Heatmap of DEGs (|Log_2_(foldchange)|> 0.5 and *p* < 0.05) between control (n = 3) and METH-treated cells (n = 3). **c** The protein–protein interaction (PPI) network of DEGs. The up-regulated genes are marked in red, while the down-regulated genes are marked in blue. The greater the difference in expression, the darker the color. The size of nodes represents the number of edges of the node; the larger the size, the greater the number of edges. **d** 10 hub genes were identified with the MCC algorithm, using cytoHubba. The score is indicated in red color. Darker color indicates a higher score

In Jurkat cells, RNA-seq results showed that there were 386 significantly different genes after METH treatment. Among them, 239 genes were upregulated, and 147 genes were downregulated (Fig. 2a). And METH treatment group and control group were mainly clustered and showed distinct patterns (Fig. 2b). Next, the PPI network of differentially expressed genes (DEGs) and hub genes were shown in Fig. 2c, d. To analyze the biological classification of DEGs, we performed a functional enrichment analysis. Importantly, we found that DEGs were enriched in sterol metabolic processes, mainly cholesterol biosynthetic processes, and maybe localized in the endoplasmic reticulum (Additional file 3: Figure S3A–D). In addition, DEGs were also enriched in oxidoreductase activity and the AMPK signaling pathway (Additional file 3: Figure S3C, D). Next, we obtained similar results from enrichment analysis of hub genes (Additional file 3: Figure S3E–H). A list of these GO and KEGG pathways of interest as well as genes were shown in Additional file 6: Table S1.

**Fig. 3:**
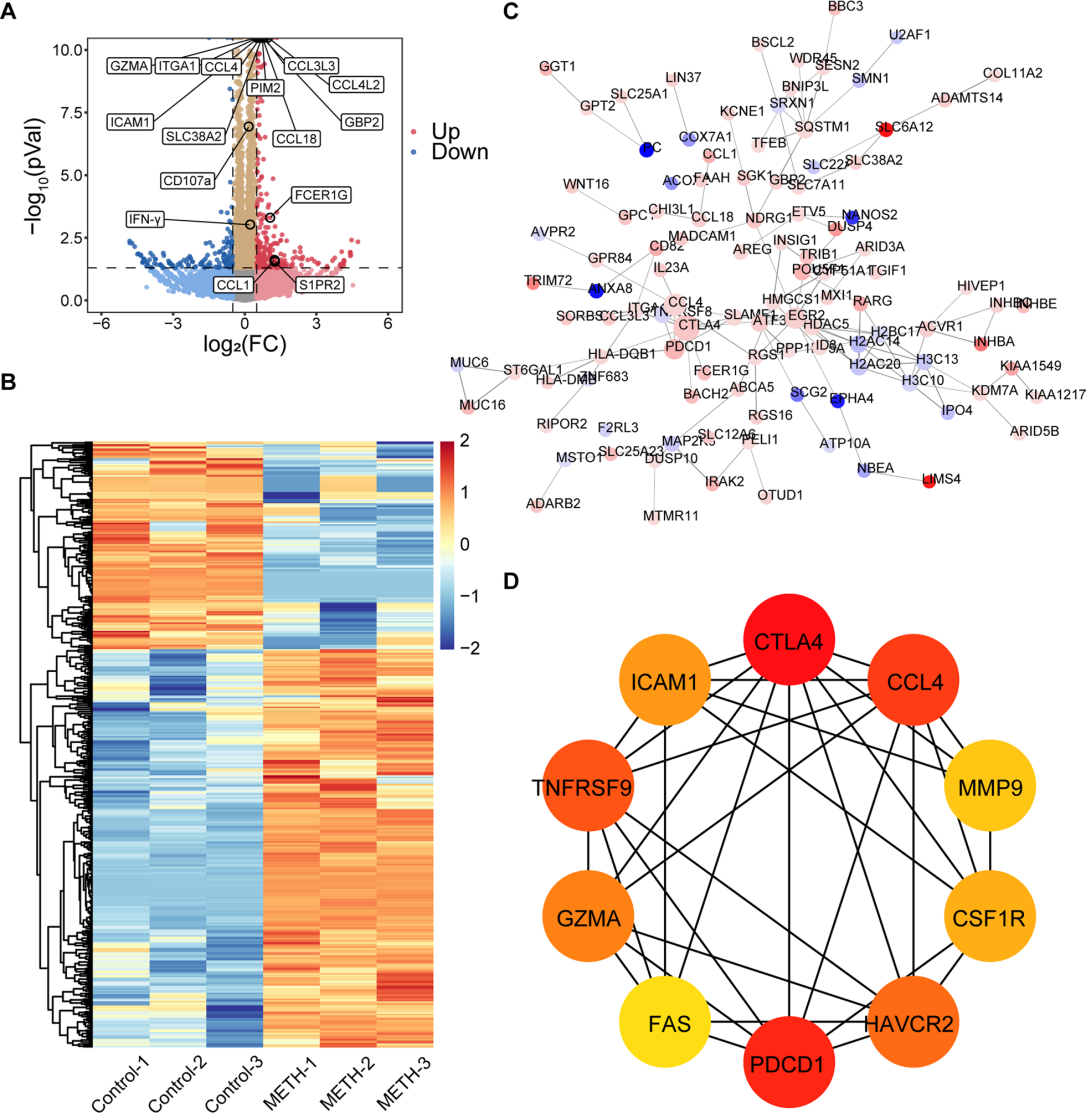
Identification of differentially expressed genes (DEGs) in NK-92 cells with methamphetamine treatment. **a** Volcano plot showing DEGs (|Log_2_(foldchange)|> 0.5 and *p* < 0.05) between control (n = 3) and METH-treated cells (n = 3). The top 10 DEGs sorted by *p* value and genes of interest were shown in the plot. **b** Heatmap of DEGs (|Log_2_(foldchange)|> 0.5 and *p* < 0.05) between control (n = 3) and METH-treated cells (n = 3). **c** The protein–protein interaction (PPI) network of DEGs. The up-regulated genes are marked in red, while the down-regulated genes are marked in blue. The greater the difference in expression, the darker the color. The size of nodes represents the number of edges of the node; the larger the size, the greater the number of edges. **d** 10 hub genes were identified with the MCC algorithm, using cytoHubba. The score is indicated in red color. Darker color indicates a higher score

In NK-92 cells, 432 DEGs were identified, including 284 up-regulated genes and 148 down-regulated genes, as shown in the volcano map and heat map (Fig. 3a, b). The PPI network of DEGs and hub genes and most dense connected regions were obtained by Cytoscape (Fig. 3c, d). By GO analysis, we found those DEGs were mainly enriched in ERK1 and ERK2 cascade, response to cytokines and cell migration (Additional file 4: Figure S4A–C). In addition, DEGs were also enriched in leukocyte chemotaxis, cytokine activity, chemokine activity, cytokine-cytokine receptor interaction (Additional file 4: Figure S4A–C), and negative regulation of cell population proliferation (Fig. 5b). However, the results did not show a significant cellular component enrichment. Next, we also obtained similar results from enrichment analysis of hub genes (Additional file 4: Figure S4D–G). The main cellular components enrichment of hub genes are the external side of the plasma membrane and immunological synapse (Additional file 4: Figure S4E). A list of these GO and KEGG pathways of interest as well as genes were shown in Additional file 6: Table S2.

**Fig. 4:**
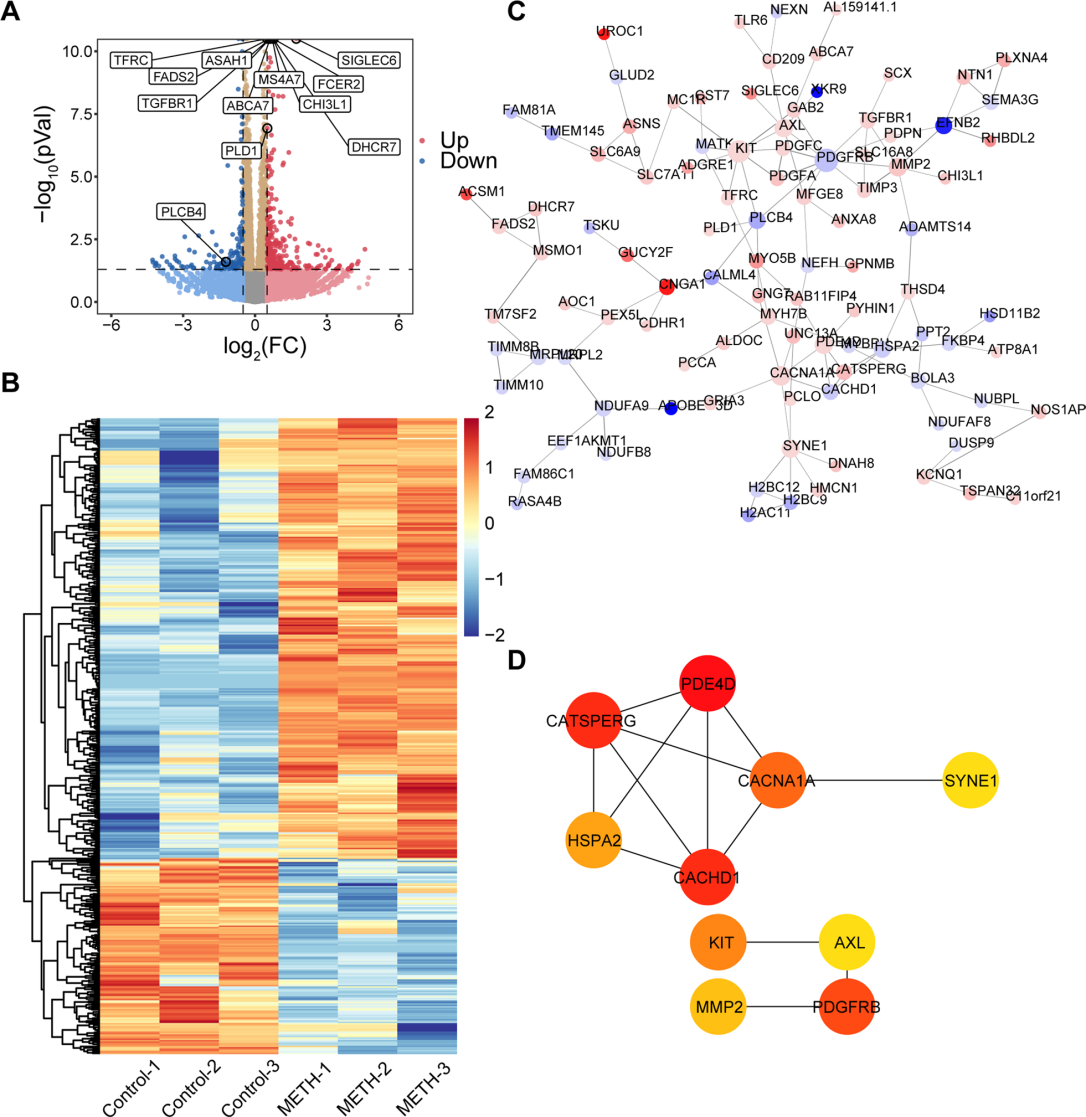
Identification of differentially expressed genes (DEGs) in THP-1 cells with methamphetamine treatment. **a** Volcano plot showing DEGs (|Log_2_(foldchange)|> 0.5 and *p* < 0.05) between control (n = 3) and METH-treated cells (n = 3). The top 10 DEGs sorted by *p* value and genes of interest were shown in the plot. **b** Heatmap of DEGs (|Log_2_(foldchange)|> 0.5 and *p* < 0.05) between control (n = 3) and METH-treated cells (n = 3). **c** The protein–protein interaction (PPI) network of DEGs. The up-regulated genes are marked in red, while the down-regulated genes are marked in blue. The greater the difference in expression, the darker the color. The size of nodes represents the number of edges of the node; the larger the size, the greater the number of edges. **d** 10 hub genes were identified with the MCC algorithm, using cytoHubba. The score is indicated in red color. Darker color indicates a higher score

In THP-1 cells, 450 DEGs were identified, including 311 up-regulated genes and 139 down-regulated genes, as shown in the volcano map and heat map (Fig. 4a, b). The PPI network of DEGs and hub genes and most dense connected regions were obtained by Cytoscape (Fig. 4c, d). Next, we performed a functional enrichment analysis of DEGs. However, no significant biological classification enrichment of DEGs was found after analysis by GO and KEGG. We only found that DEGs were enriched in cellular components on the external side of the plasma membrane and calcium channel complex (Additional file 5: Figure S5B). Therefore, then we performed enrichment analysis using Metascape instead. We found that DEGs were mainly enriched in response to copper ion, cell–cell adhesion, small molecule biosynthetic process, positive regulation of protein autophosphorylation, and regulation of cell activation, etc. (Additional file 5: Figure S5A). Next, we performed GO and KEGG enrichment analyses of hub genes and found that they were mainly associated with calcium ion transport and regulation of calcium channel (Additional file 5: Figure S5C–F). A list of these GO and KEGG pathways of interest as well as genes were shown in Additional file 6: Table S3.

### Combined enrichment analysis of DEGs of three types of immune cells

**Fig. 5:**
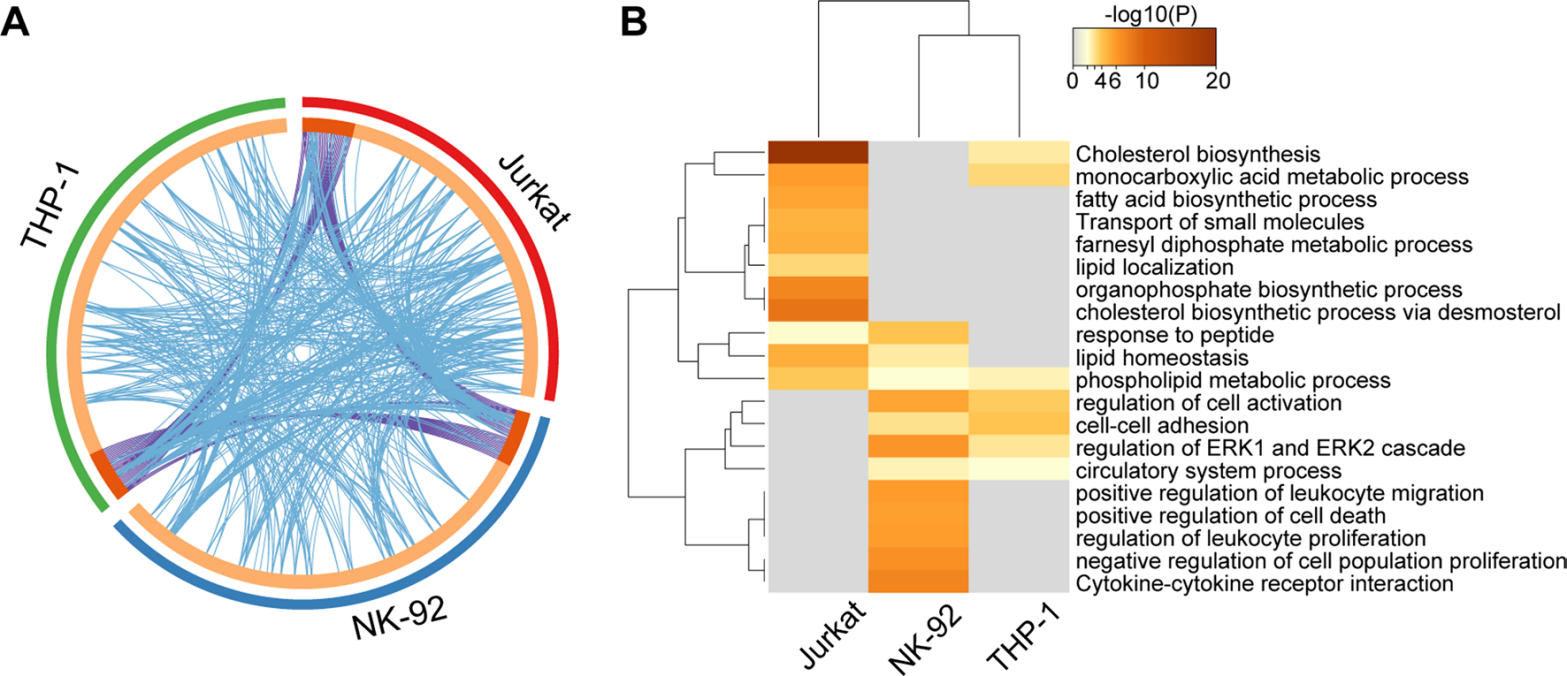
Combined analysis of three types of immune cells. **a** Circos plot showing shared differentially expressed genes (DEGs) between three types of immune cells. The shared genes are linked by purple lines, and the different genes falling into the same term are linked by blue lines. **b** Heatmap showing enrichment of DEGs of Jurkat, NK-92 and THP-1 cell. The color from gray to brown indicates high to low *p* values, respectively

To find the potential common biological classification of METH affecting the three immune cells, we performed a combined analysis of DEGs from three cell types using Metascape. We found 3 DEGs shared by the three cell types, 22 genes shared by Jurkat and NK-92, 21 by Jurkat and THP-1, and 22 by NK-92 and THP-1 (Fig. 5a). Next, we found that the three cell types had similar response patterns to METH treatment in certain biological processes or pathways. As shown in Fig. 5b, DEGs of the three cell types are co-enriched in the phospholipid metabolic process. DEGs of Jurkat and THP-1 co-enriched in cholesterol biosynthesis and monocarboxylic acid metabolic process. DEGs of Jurkat and NK-92 co-enriched in response to peptide lipid homeostasis. And DEGs of NK-92 and THP-1 co-enriched in the regulation of cell activation, cell–cell adhesion, regulation of ERK1 and ERK2 cascade, and circulatory system process (Fig. 5b).

### qPCR validation

**Fig. 6:**
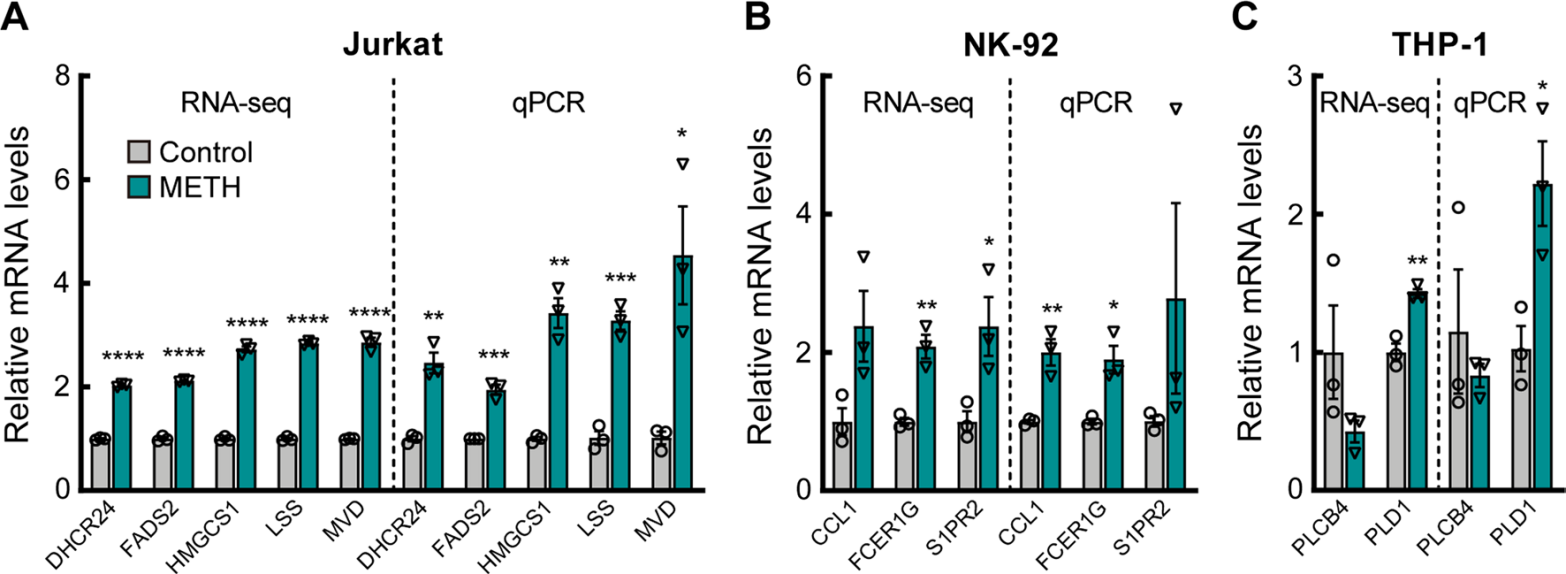
Comparison of qPCR validation and RNA-seq results of DEGs enriched in important biological processes and pathways. **a** DEGs from Jurkat cells. **b** DEGs from NK-92 cells. **c** DEGs from THP-1 cells. Data represent mean ± SEM from three distinct biological replicates (*p < 0.05; **p < 0.01; ***p < 0.001; ****p < 0.0001, compared with control)

We selected several DEGs that were enriched in important biological processes and pathways for qPCR validation. Based on the RNA-seq enrichment results, we selected a part of genes enriched in the cholesterol biosynthesis process, cytokine-cytokine receptor interaction, negative regulation of cell population proliferation, and monocarboxylic acid metabolism process for validation. These genes were generally expressed in higher relative amounts than others and had lower *p* values. Figure 6a showed the mRNA relative expression of *MVD*, *HMGCS1*, *FADS2*, *LSS*, and *DHCR24*, enriched in cholesterol biosynthesis in Jurkat cells. Figure 6b showed the mRNA relative expression of *CCL1* and *FCER1G* which were enriched in cytokine-cytokine receptor interaction and *S1PR2* which was enriched in negative regulation of cell population proliferation in NK-92 cells. Figure 6c showed the mRNA relative expression of *PLCB4* and *PLD1* which were enriched in the monocarboxylic acid metabolic process in THP-1 cells. The trends of expression levels of DEGs were consistent with the RNA-seq results, indicating favorable reliability of RNA-seq.

## Discussion

Immune cells are an important part of the immune system, and an increasing number of studies have found them to be impaired in METH abuse [23, 24, 32]. In this study, we constructed models of METH-treated cells of three common immune cells in vitro and evaluated the effects of METH in the resting state of immune cells. We found that T cells, NK cells and monocytes all showed varying degrees of response. Indeed, similar to some of the previous findings, METH affected the proliferation of all three cell types to some extent and led to apoptosis in NK cells. At the transcriptome level, T cells mainly showed disorders of cholesterol metabolism, NK cells showed activation of ERK1/ERK2 cascade and cytokine-related responses, and THP-1 cells showed disorders of calcium ion transmembrane transport. To some extent, the above results may reflect the adverse effects of METH abuse on immune cells.

In this study, we treated these three common immune cells with 250 μM METH for 24 h and found that there was no significant effect on cell viability. And this treatment concentration and time were used in subsequent transcriptome tests. This is similar to the drug concentration in blood, urine or tissues of people who use METH, about 2 μM to 600 μM [19, 33–35]. The concentration we used was higher compared to other similar studies we mentioned before, and they were mostly around 100 μM. This may cause our results to be partially different from other studies. However, binge states may occur during drug use over time and lead to an escalation of drug abuse [36, 37]. This makes it meaningful to use METH at a higher concentration without cytotoxicity in this experiment.

T cells are an important component of the immune system, regulating multiple aspects of adaptive immunity, including responses to pathogens, allergens and tumors [38]. We found that METH abuse led to changes of steroid metabolic processes in Jurkat cells, mainly cholesterol metabolism (Additional file 3: Figure S3A, E). The results were verified by qPCR (Fig. 6a), and several genes encoding key enzymes in the cholesterol synthesis pathway were significantly upregulated [39]. Interestingly, some of these genes may play a protective role in some cases. *MVD* encodes mevalonate diphosphate decarboxylase, and catalyzes the conversion of mevalonate pyrophosphate into isopentenyl pyrophosphate in the early steps of cholesterol biosynthesis [40]. *HMGCS1* (3-hydroxy-3-methylglutaryl-CoA synthase 1) is also involved in the mevalonate pathway. It is a regulatory node in cholesterol biosynthesis, and may promote cell proliferation [41]. *FADS2* (fatty acid desaturase 2) may be involved in the coordination of inflammatory responses and metabolic homeostasis [42]. *LSS* (lanosterol synthase) catalyzes the conversion of (S)-2,3 oxidosqualene to lanosterol and catalyzes the first step in the biosynthesis of cholesterol, and may play a protective role in the early stages of oxidative stress [43]. *DHCR24* (24-dehydrocholesterol reductase) catalyzes the reduction of the delta-24 double bond of sterol intermediates during cholesterol biosynthesis, and may alleviate oxidative stress and reduce apoptosis [44, 45]. METH always leads to oxidative stress. For example, studies showed that METH caused mitochondrial dysfunction in microglia or nerve cells, increased oxidative stress, and led to cell apoptosis [46, 47]. In our results, DEGs were also enriched in molecular functions such as oxidoreductase activity and NADP binding, and the AMPK signaling pathway (Additional file 3: Figure S3C, D). The AMPK signaling pathway is thought to be related to oxidative stress [48]. These results suggest that METH may lead to oxidative stress in T cells. Therefore, the upregulation of the cholesterol synthesis process and related genes may be the protective response of T cells to METH stimulation.

Cholesterol is a class of steroids that forms the basic structure of cell membranes and is also a metabolic intermediate and signaling factor involved in the coordination of many biological processes [49]. However, excess cholesterol may lead to adverse effects. Several studies have shown an association between cholesterol and T-cell immunosuppression. In breast cancer, elevated cholesterol levels were associated with tumor recurrence and metastasis, and the oxysterol metabolite of cholesterol, 27-hydroxycholesterol, led to a decrease in the number of cytotoxic CD8^+^ T lymphocytes and promoted immunosuppression [50]. It was found that lower levels of cholesterol in an IL-9-secreting Tc9 cell formed by CD8^+^ T cells enhanced its antitumor effect and cell differentiation [51]. In addition, in the tumor microenvironment, cholesterol increased endoplasmic reticulum (ER) stress leading to high levels of immune checkpoint expression and cellular exhaustion in CD8^+^ T cells, and in contrast, its anti-tumor activity could be effectively restored by lowering cellular cholesterol [52]. Conversely, however, it had also been shown that antitumor effects were enhanced by increasing plasma membrane cholesterol levels. The researchers found that the cholesterol levels were markedly increased in activated CD8^+^ T cells, and cholesterol biosynthesis and transport pathways were upregulated. Inhibition of cholesterol synthesis and transport led to decreased granule and cytokine production. Elevated CD8^+^ T cell plasma membrane cholesterol levels through inhibition of ACAT1, a key cholesterol esterase, led to enhanced T cell receptor clustering, as well as more efficient signal transduction and immunological synapse formation [53]. In our results, there was no significant difference in *ACAT1*, but *ACAT2* (acetyl-CoA acetyltransferase 2) was significantly upregulated (Fig. 2a). ACAT2 is an isoenzyme of ACAT1, which also produces cholesterol esters. In contrast, it has been shown that inhibition of *ACAT2* impairs the expansion of TCR-driven CD4^+^ and CD8^+^ T cells [54]. To some extent, our results also suggested that T cells may be activated. In addition, cholesterol metabolism is involved in autophagy, inflammation and apoptosis, and is associated with ER stress, and severe ER stress may lead to apoptosis [49]. Moreover, most of the cholesterol is located in membrane structures called lipid rafts on the cell membrane and is involved in receptor composition and protein signaling [55], such as TCR signaling, which is sensitive to cholesterol content [56]. Therefore, we hypothesize that METH causes oxidative stress, and further lead to cholesterol metabolism disruption and leads to dysfunction, decreased cellular activity and apoptosis in T cells. However, as far as we know, this has not been hypothesized in METH-induced T-cell impairment and therefore requires further study.

NK cells are a class of innate lymphocytes that can directly kill target cells and play a key role in processes such as antiviral and antitumor [57]. We showed that METH significantly activated NK cells at the transcriptional level, mainly in response to cytokines, cell chemotaxis and cell migration, possibly mediated by ERK1 and ERK2 cascade (Additional file 4: Figure S4A–C). ERK1 and ERK2 cascade is a MAPK signaling pathway that is normally activated by a variety of factors, including cytokines, hormones, and cellular stress and is associated with cell proliferation, differentiation, cell migration, stress, inflammatory response, apoptosis, and other processes [58–60]. Similar to previous in vivo results in animals, the study found that METH increased the expression level of CD107a, TNF-α and IFN-γ in NK cells [18]. In our results, CD107a and IFN-γ were significantly upregulated, although the fold change was small (Fig. 3a). The results of qPCR validation showed that *CCL1* was significantly upregulated (Fig. 6b). Studies show that activated NK cells can produce CCL1, which may recruit other immune cells during the immune response and mediate the inflammatory response [61]. The expression of *FCER1G* (Fc epsilon receptor Ig) was increased (Fig. 6b), and may be related to the increased regulatory activity of NK cells [62]. And the expression of *S1PR2* (sphingosine-1-phosphate receptor 2) was increased (Fig. 6b), and may be involved in the ERK1/2 signaling pathway [63]. In addition, DEGs were also enriched in response to cytokine, cell migration and chemotaxis (Additional file 4: Figure S4A–C). These results all suggested that NK cells were activated. However, sustained activation may lead to depletion of immune cells [64]. Our results showed that METH led to an increased proportion of NK-cell apoptosis (Fig. 1c). It suggests that METH may lead to NK-cell immune dysregulation and death through ERK1 and ERK2 cascade.

Monocytes are also a key part of the immune system and are often thought to be a common source of dendritic cells and macrophages [65]. We found that METH use is associated with calcium ion transmembrane transport and calcium channel activity in THP-1 cells (Additional file 5: Figure S5C–E). Calcium ion is an intracellular second messenger involved in the regulation of various biological processes such as proliferation, apoptosis and immune responses, and various diseases such as autoimmune diseases and viral infections [66]. For example, in macrophages, calcium ions can regulate the production of TNF-α [67] and nitric oxide [68]. Moreover, calcium ion promoted DCs activation and maturation in vitro and play a role in responses to TLR ligands, bacteria, and microbial toxins [69]. Therefore, METH-induced calcium ion disruption may lead to functional impairment of THP-1 cells. In the qPCR validation results, PLCB4 (phospholipase C beta 4) and PLD1 (phospholipase D1) were changed (Fig. 6c). They can promote cell signal transduction, second messenger generation, intracellular calcium ions release, protein kinase C activation, and cause cell activation [70, 71]. In addition, DEGs were also enriched in cell–cell adhesion (Additional file 5: Figure S5A), which may be involved in the pro-inflammatory response [72]. Therefore, the above results suggest that METH may cause THP-1 cell activation and cell function impairment.

We also found some common responses of different immune cells to the effects of METH. Similar to Jurkat, METH disrupts lipid homeostasis in NK-92 cells (Fig. 5b). Recent studies have found that increased lipid metabolism impaired NK-cell function in aggressive B-cell lymphoma [73], suggesting that METH-induced lipid disruption may also lead to NK-cell dysfunction. Likewise, THP-1 showed disruption of cholesterol biosynthesis (Fig. 5b). It has been shown that there was an association between cholesterol biosynthesis and impaired monocytes cytokine production in the context of human lower respiratory tract infections [74]. It is worth noting that all three cell types showed disturbance of the phospholipid metabolic process after METH treatment (Fig. 5b). Phospholipids comprise a large number of lipids that define cells and organelles by forming membrane structures, and their complex metabolism represents a highly controlled cellular signaling network, which studies have shown to be critical for establishing an effective immune response [75]. Once the process of phospholipid metabolism is disturbed, it will cause immune cell dysfunction, and this may be a common pathway for METH to mediate adaptive immunity and innate immune impairment. Interestingly, METH also caused disruption of phospholipid metabolism in the mouse brain [76], suggesting a potential common pathway for METH injury to the organism.

There are several limitations in our present study. First, we lack validation of protein expression levels of key differential genes. Second, the subsequent functional studies are critical but absent in the present work. Third, the mechanisms of how METH leads to these immune cell responses remain unclear. Fourth, the studies were performed in vitro and do not well simulate the complex environment in vivo. In addition, this study did not identify and analyze the subsets of immune cells. Therefore, the next in-depth study is necessary.

## Conclusion

We explored the response of immune cells to METH abuse, importantly at the transcriptome level. The transcriptomic changes are mostly proposed for the first time. These results provide new insights into the potential effects by which METH injures the human immune system.

## Supplementary Information

**Additional file 1**. **Figure S1. **Methamphetamine inhibited immune cell viability and promoted apoptosis. CCK-8 assay was used to assess Jurkat cell (A), NK-92 cell (B) and THP-1 cell (C) viability after treatment with different METH concentrations and different treatment time. Data represent mean ± SEM from three to seven independent experiments (*p < 0.05; **p < 0.01; ***p < 0.001; ****p < 0.0001, compared with 0 μM).

**Additional file 2**. **Figure S2**. Flow cytometry results of apoptosis of immune cells. The representative results showed the apoptosis of different immune cells after 0-1000 μM methamphetamine treatment for 12 h, 24 h, and 48 h. The results were representative of at least three independent experiments.

**Additional file 3**. **Figure S3. **GO and KEGG pathway functional enrichment analysis of Jurkat cells. (A-D) GO and KEGG pathway functional enrichment analysis of DEGs. (E-H) GO and KEGG pathway functional enrichment analysis of hub genes. Top 10 sorted by *p* value of GO terms or KEGG pathways were shown. GO: Gene Ontology; KEGG: Kyoto Encyclopedia of Genes and Genomes; DEGs: differentially expression genes.

**Additional file 4**. **Figure S4. **GO and KEGG pathway functional enrichment analysis of NK-92 cells. (A-C) GO and KEGG pathway functional enrichment analysis of DEGs. (D-G) GO and KEGG pathway functional enrichment analysis of hub genes. Top 10 sorted by *p* value of GO terms or KEGG pathways were shown. GO: Gene Ontology; KEGG: Kyoto Encyclopedia of Genes and Genomes; DEGs: differentially expression genes.

**Additional file 5**. **Figure S5. **Metascape, GO and KEGG pathway functional enrichment analysis of THP-1 cells. (A) Metascape enrichment analysis of DEGs. (B) GO cellular component enrichment analysis of DEGs. (C-F) GO and KEGG pathway functional enrichment analysis of hub genes. Top 20 sorted by *p* value of Metascape analysis and top 10 sorted by *p* value of GO terms or KEGG pathways were shown. GO: Gene Ontology; KEGG: Kyoto Encyclopedia of Genes and Genomes; DEGs: differentially expression genes.

**Additional file 6**. **Table S1.** GO and KEGG pathways of interest in Jurkat cells. **Table S2.** GO and KEGG pathways of interest in NK-92 cells. **Table S3.** GO and KEGG pathways of interest in THP-1 cells.

## Data Availability

The datasets generated and/or analysed during the current study are available in the Gene Expression Omnibus repository, Accession Number GSE201007 (https://www.ncbi.nlm.nih.gov/geo/query/acc.cgi?acc=GSE201007).

## Abbreviations

CCK-8: Cell Counting Kit 8
DCs: Dendritic cells
DEGs: Differentially expressed genes
ER: Endoplasmic reticulum
GEO: Gene Expression Omnibus
GO: Gene Ontology
HCV: Hepatitis C virus
HIV: Human immunodeficiency viruses
KEGG: Kyoto Encyclopedia of Genes and Genomes
METH: Methamphetamine
NK: Nature killing
PBS: Phosphate-buffered saline
PI: Propidium iodide
PPI: Protein–protein interaction
qPCR: Quantitative real-time PCR
RNA-seq: RNA sequencing
