# Supplementary figures and images for: Effects and associated transcriptomic landscape changes of methamphetamine on immune cells
Source: BMC Med Genomics. 2022 Jun 28;15:144. doi: 10.1186/s12920-022-01295-9 (PMC9241331; doi:10.1186/s12920-022-01295-9)

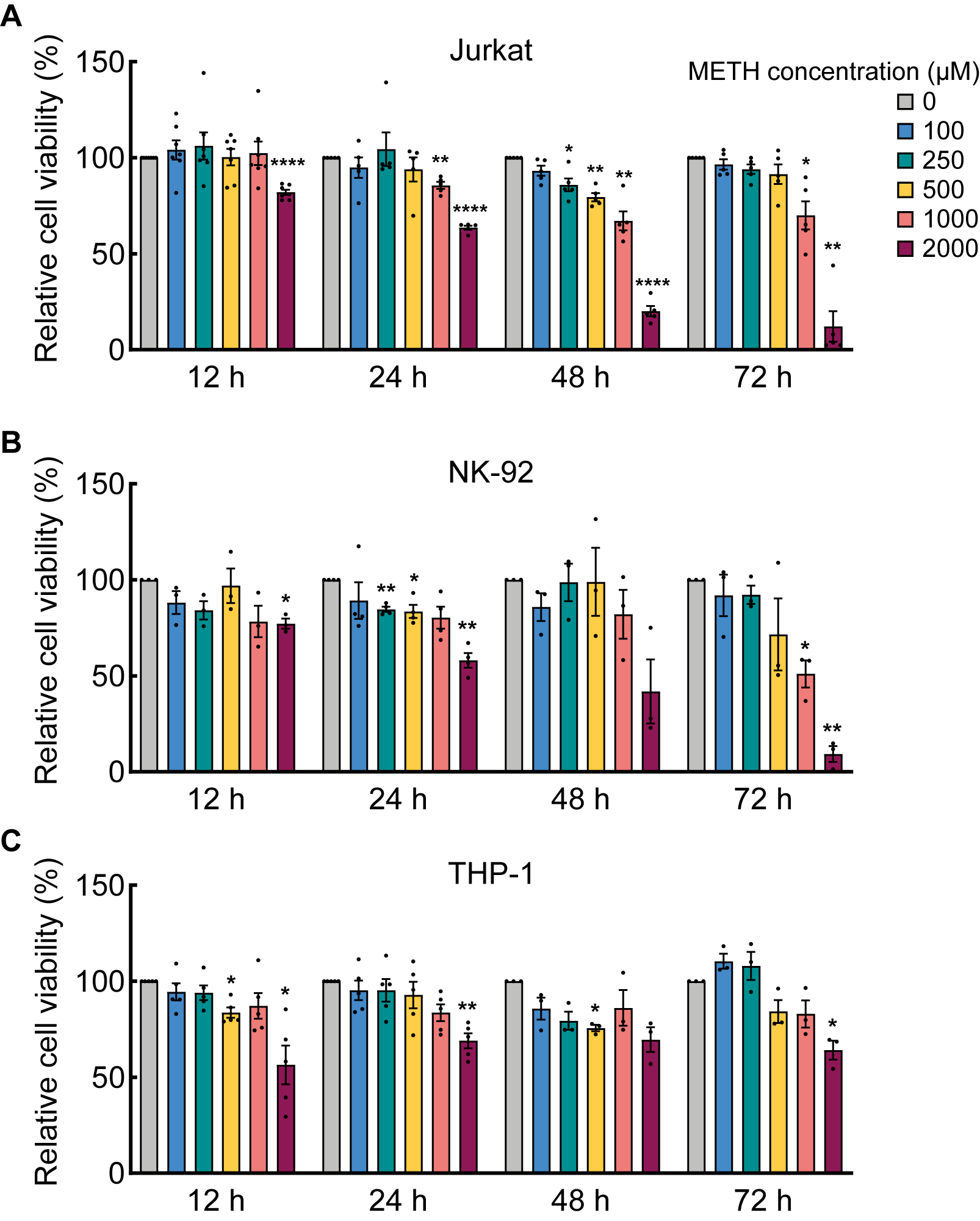

Supplement: Supplementary file 1 — Additional file 1. Figure S1. Methamphetamine inhibited immune cell viability and promoted apoptosis. CCK-8 assay was used to assess Jurkat cell (A), NK-92 cell (B) and THP-1 cell (C) viability after treatment with different METH concentrations and different treatment time. Data represent mean ± SEM from three to seven independent experiments (*p < 0.05; **p < 0.01; ***p < 0.001; ****p < 0.0001, compared with 0 μM). [file 12920_2022_1295_MOESM1_ESM.tif]

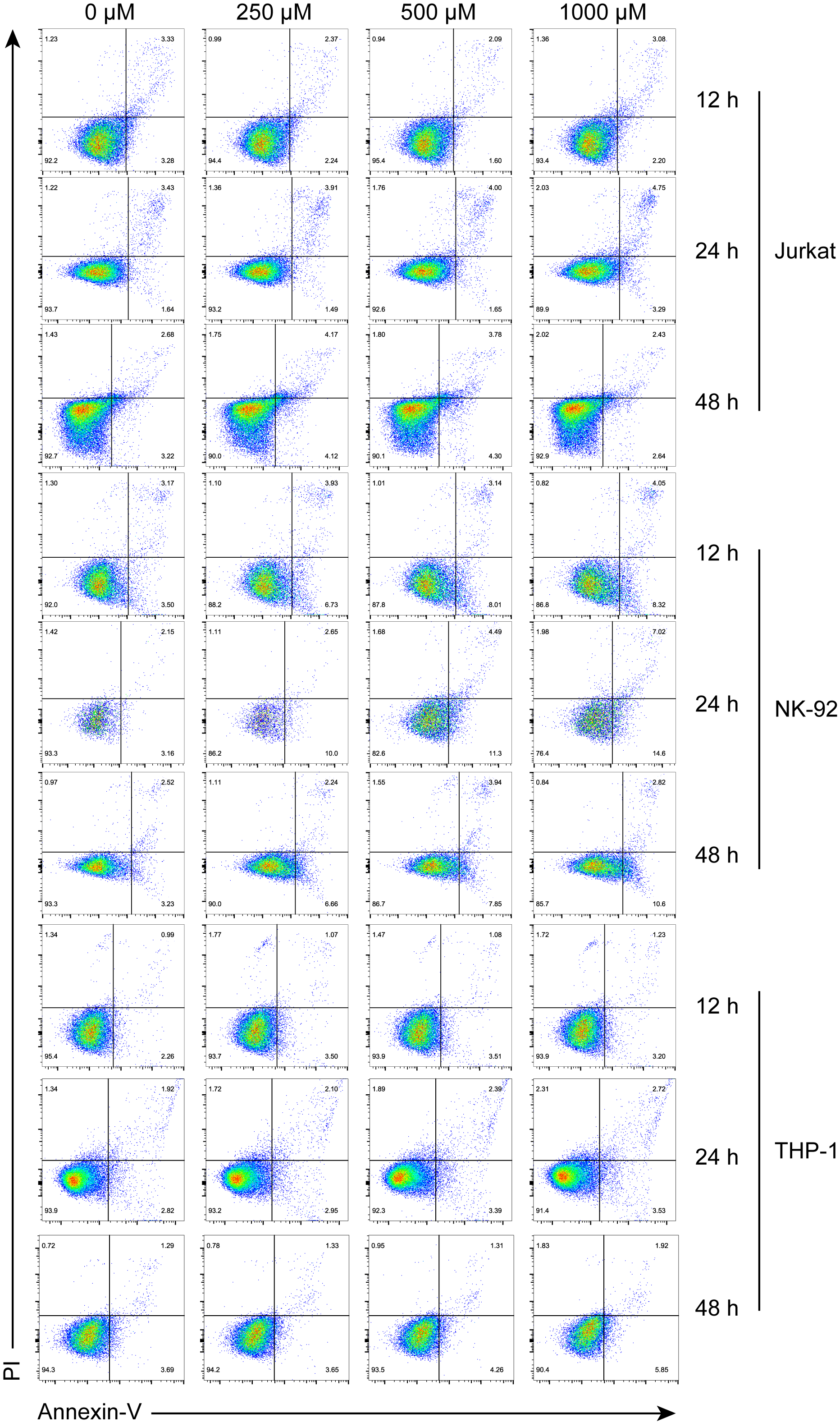

Supplement: Supplementary file 2 — Additional file 2. Figure S2. Flow cytometry results of apoptosis of immune cells. The representative results showed the apoptosis of different immune cells after 0-1000 μM methamphetamine treatment for 12 h, 24 h, and 48 h. The results were representative of at least three independent experiments. [file 12920_2022_1295_MOESM2_ESM.tif]

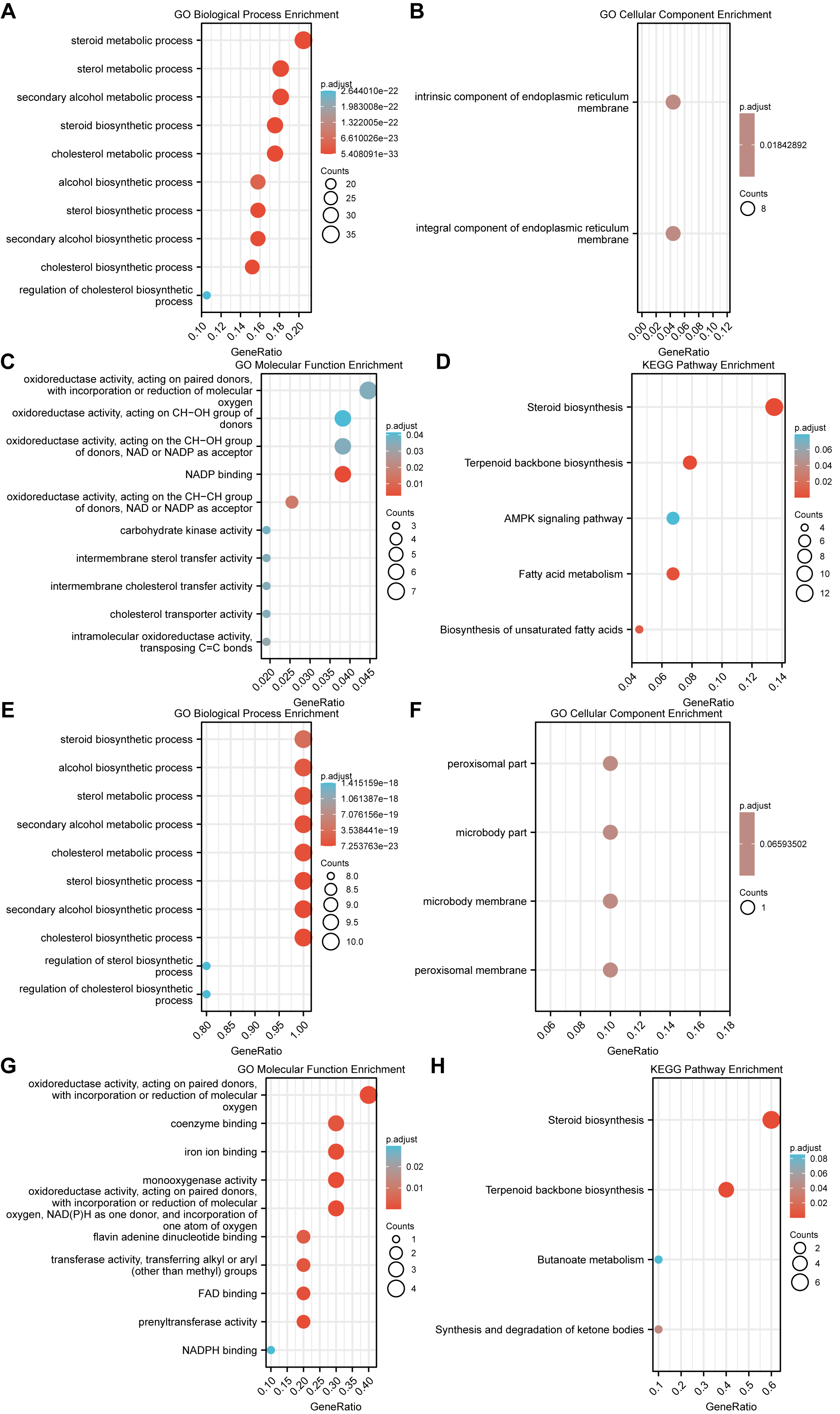

Supplement: Supplementary file 3 — Additional file 3. Figure S3. GO and KEGG pathway functional enrichment analysis of Jurkat cells. (A-D) GO and KEGG pathway functional enrichment analysis of DEGs. (E-H) GO and KEGG pathway functional enrichment analysis of hub genes. Top 10 sorted by p value of GO terms or KEGG pathways were shown. GO: Gene Ontology; KEGG: Kyoto Encyclopedia of Genes and Genomes; DEGs: differentially expression genes. [file 12920_2022_1295_MOESM3_ESM.tif]

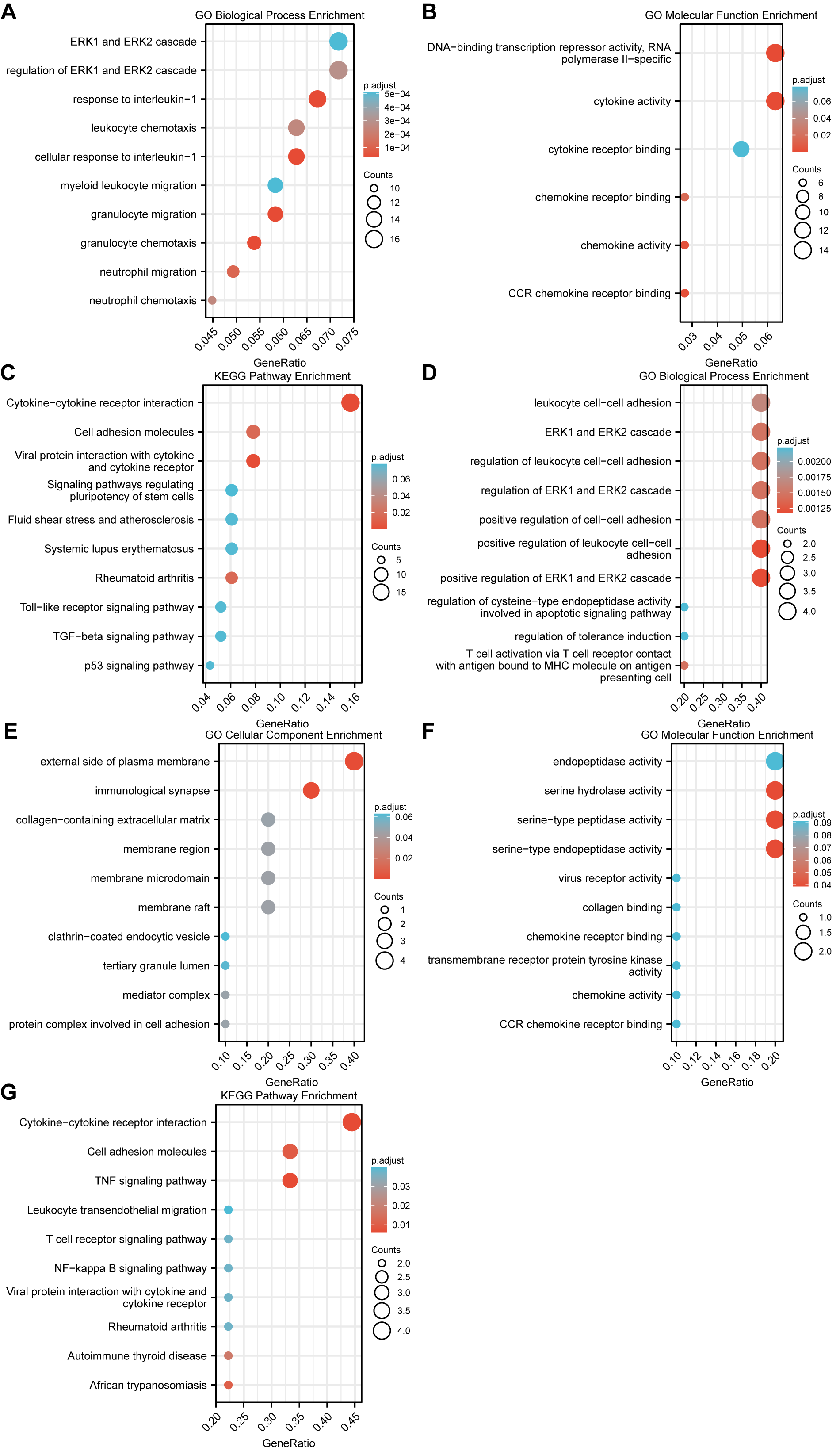

Supplement: Supplementary file 4 — Additional file 4. Figure S4. GO and KEGG pathway functional enrichment analysis of NK-92 cells. (A-C) GO and KEGG pathway functional enrichment analysis of DEGs. (D-G) GO and KEGG pathway functional enrichment analysis of hub genes. Top 10 sorted by p value of GO terms or KEGG pathways were shown. GO: Gene Ontology; KEGG: Kyoto Encyclopedia of Genes and Genomes; DEGs: differentially expression genes. [file 12920_2022_1295_MOESM4_ESM.tif]

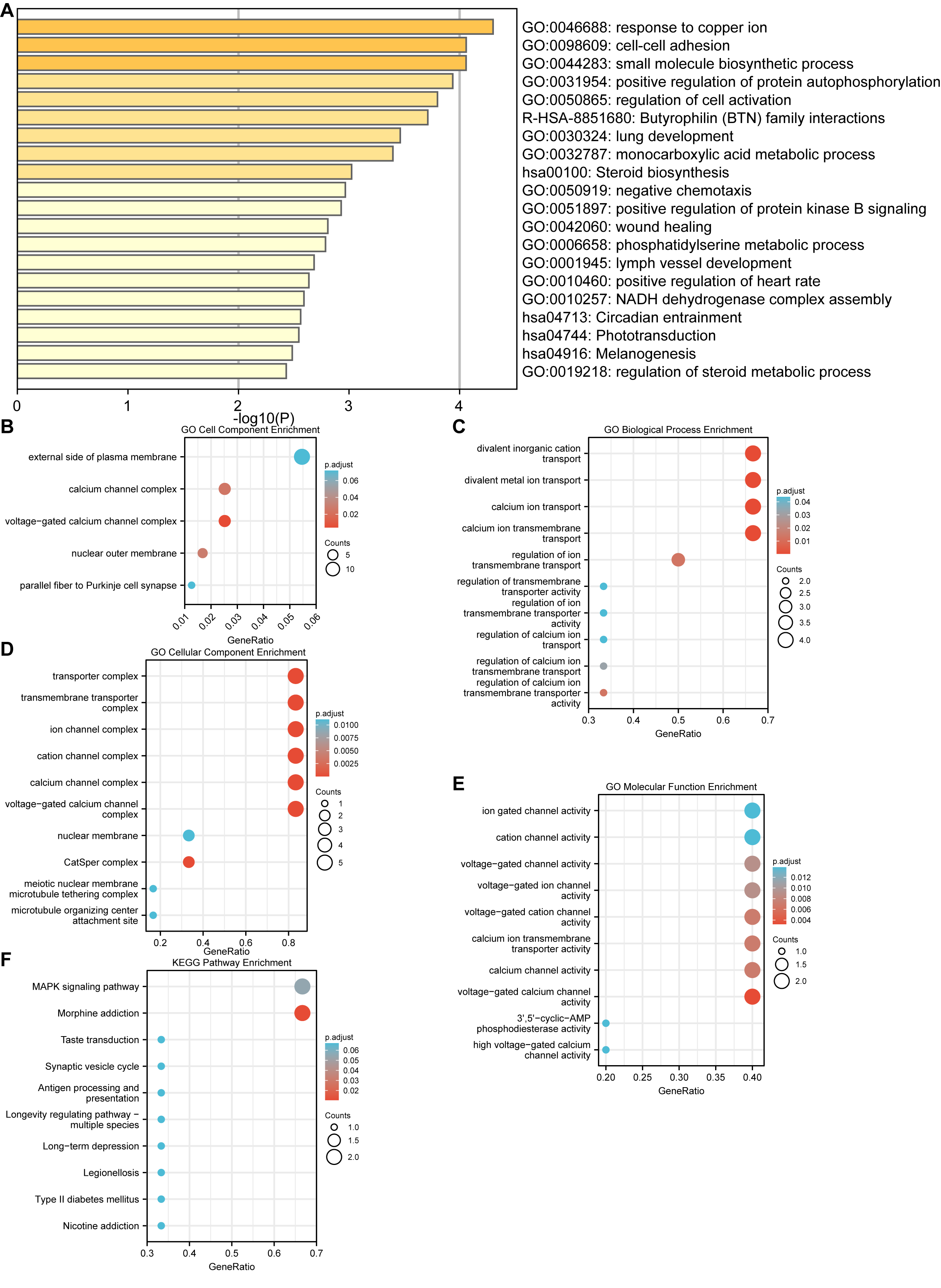

Supplement: Supplementary file 5 — Additional file 5. Figure S5. Metascape, GO and KEGG pathway functional enrichment analysis of THP-1 cells. (A) Metascape enrichment analysis of DEGs. (B) GO cellular component enrichment analysis of DEGs. (C-F) GO and KEGG pathway functional enrichment analysis of hub genes. Top 20 sorted by p value of Metascape analysis and top 10 sorted by p value of GO terms or KEGG pathways were shown. GO: Gene Ontology; KEGG: Kyoto Encyclopedia of Genes and Genomes; DEGs: differentially expression genes. [file 12920_2022_1295_MOESM5_ESM.tif]
