# Supplementary material for: Effects and associated transcriptomic landscape changes of methamphetamine on immune cells
Source: BMC Med Genomics. 2022 Jun 28;15:144. doi: 10.1186/s12920-022-01295-9 (PMC9241331; doi:10.1186/s12920-022-01295-9)
Supplement: Supplementary file 6 — Additional file 6. Table S1. GO and KEGG pathways of interest in Jurkat cells. Table S2. GO and KEGG pathways of interest in NK-92 cells. Table S3. GO and KEGG pathways of interest in THP-1 cells. [file 12920_2022_1295_MOESM6_ESM.docx]

**Table S1. GO and KEGG pathway of interest in Jurkat.**

| **Description** | **Differentially expressed genes** |
| --- | --- |
| Cholesterol biosynthetic process | *ACAT2, ACLY, CYP51A1, DHCR7, DHCR24, FASN, FDFT1, FDPS, HMGCR, HMGCS1, IDI1, INSIG1, LSS, MVD, MVK, MSMO1, SC5D, SCD, SQLE, SREBF2, TM7SF2, ABCG1, EBP, NSDHL, HSD17B7, ELOVL6* |
| Integral component of endoplasmic reticulum membrane | *CLN3, DHCR7, INSIG1, SCD, SREBF2, TM7SF2, ELOVL6, EMC6* |
| Oxidoreductase activity, acting on paired donors, with incorporation or reduction of molecular oxygen | *CYP51A1, FADS1, MSMO1, SC5D, SCD, SQLE, FADS2* |
| Oxidoreductase activity, acting on the CH-OH group of donors, NAD or NADP as acceptor | *FASN, HMGCR, IDH1, NSDHL, HSD17B14, HSD17B7* |
| AMPK signaling pathway | *FASN, HMGCR, LEPR, PFKFB4, PPP2R2B, SCD* |

**Table S2. GO and KEGG pathway of interest in NK-92.**

| **Description** | **Differentially expressed genes** |
| --- | --- |
| ERK1 and ERK2 cascade | *ATF3, CHI3L1, CSF1R, DUSP4, ICAM1, LGALS9, CCL1, CCL3L1, CCL4, CCL18, SLAMF1, DUSP10, HAVCR2, SCIMP, CCL4L1, CCL3L3* |
| Response to interleukin-1 | *CEBPB, CHI3L1, EGR1, GBP2, ICAM1, IRAK2, LGALS9, CCL1, CCL3L1, CCL4, CCL18, SQSTM1, PELI1, CCL4L1, CCL3L3* |
| Leukocyte chemotaxis | *LYST, CMKLR1, FCER1G, ITGA1, LGALS9, CCL1, CCL3L1, CCL4, CCL18, SCG2, RIPOR2, IL23A, CCL4L1, CCL3L3* |
| Myeloid leukocyte migration | *CMKLR1, FCER1G, ITGA1, PECAM1, CCL1, CCL3L1, CCL4, CCL18, SCG2, RIPOR2, IL23A, CCL4L1, CCL3L3* |
| Cytokine activity | *AREG, INHBA, INHBC, CCL1, CCL3L1, CCL4, CCL18, SCG2, EBI3, IL24, IL23A, INHBE, CCL4L1, CCL3L3* |
| Chemokine activity | *CCL1, CCL3L1, CCL4, CCL18, CCL4L1, CCL3L3* |
| Cytokine-cytokine receptor interaction | *ACVR1, FAS, TNFRSF8, CSF1R, TNFRSF9, INHBA, INHBC, CCL1, CCL3L1, CCL4, CCL18, CCL4L2, EBI3, IL24, IL23A, INHBE, CCL4L1, CCL3L3* |
| Negative regulation of cell population proliferation | *AVPR2, BCL6, TNFRSF8, CEBPB, CSF1R, CTLA4, TNFRSF9, INHBA, ITGA1, LGALS9, RARG, SCG2, S1PR2, TRIB1, NDRG1, IL24, PIM2, DUSP10, CRTAM, PELI1, HAVCR2, CLDN19, MIR199A1, MIR98, CCL3L3* |

**Table S3. GO and KEGG pathway of interest in THP-1.**

| **Description** | **Differentially expressed genes** |
| --- | --- |
| Cell-cell adhesion | *KIT, CEACAM6, CLDN2, NTN1, TSPAN32, PDPN, SLC7A11, PCDHGA12, CD209, CDON, PCDHGA4, CDH24, HMCN1, UBASH3B, NEXN, CDHR1* |
| Monocarboxylic acid metabolic process | *ALDOC, AOAH, ASAH1, LTC4S, PCCA, MSMO1, UCP3, FADS2, SDS, PNPLA8, CYP2W1, BCO2, ACSM1, UROC1, PLCB4, PLD1, ABHD4, ABHD12B* |
| Small molecule biosynthetic process | *ASAH1, ASNS, DHCR7, GLUD2, LTC4S, NAIP, NDUFA9, MSMO1, TM7SF2, FADS2, SDS, PNPLA8, PRTFDC1, ACSM1, SERINC5* |
| Positive regulation of protein autophosphorylation | *PDGFA, GPNMB, PDGFC, NEK10, CACNA1A, DUSP5, DUSP9, HSPA2, KIT, PDGFRB, RPS6KA2, TGFBR1, TLR6, CHI3L1, GCNT2, TIMP3, ABCA7, NAIP, TNIK, MMP2, CEACAM6, TNFRSF14, NTN1, ATP8A1, PDPN, SEMA3G, TRIB1, PLD1, SLC44A3, TP53INP1, AXL, HTR6, PLCB4, CALML4* |
| Regulation of cell activation | *AXL, EFNB2, HLA-DMA, IGLL1, PDGFA, PDGFRB, TFRC, CST7, TNFRSF14, GAB2, TSPAN32, TLR6, GPNMB, PDPN, CD209, GAL, UBASH3B, NRARP, GCNT2, CEACAM6, MDGA1, GPR68, TRIB1, RIN3* |
| Calcium ion transmembrane transport | *CACNA1A, HSPA2, PDE4D, CACHD1* |
